# Long non-coding RNA *SOX2-OT* enhances cancer biological traits via sponging to tumor suppressor miR-122-3p and miR-194-5p in non-small cell lung carcinoma

**DOI:** 10.1038/s41598-023-39000-0

**Published:** 2023-07-31

**Authors:** Fatemeh Dodangeh, Zahra Sadeghi, Parichehr Maleki, Jamshid Raheb

**Affiliations:** Department of Molecular Medicine, National Institute of Genetic Engineering and Biotechnology, Tehran, Iran

**Keywords:** Biochemistry, Biotechnology, Cancer, Chemical biology, Genetics, Molecular biology

## Abstract

The oncogenic role of long non-coding RNA SOX2 overlapping transcript (*SOX2-OT*) has been demonstrated as a miRNA decay system that sponges tumor suppressor miRNA, including miR-122-3p in glioblastoma and miR-194-5p in glioblastoma, gastric, and colorectal cancers. However, the molecular function of *SOX2-OT* remains unknown in most cancers, including lung cancer. In the current study, we aimed to evaluate the downstream regulatory function of *SOX2-OT* in A549 and Calu-3 lung cancer cell lines. We knocked down *SOX2-OT* expression using an RNA interference system, which significantly decreased expression in A549 and Calu-3 cells. The expression of down-regulating miRNAs (miR-122-3p and miR-194-5p) was evaluated, revealing increased expression of miR-122-3p and miR-194-5p. Additionally, the expression of miRNAs downstream mRNA, including FOXO1 (Forkhead Box O1) and FOXA1 (Forkhead Box O1), changed. Recently, critical roles of FOXO1 and FOXA1 proteins in pathways involved in proliferation, metastasis and apoptosis have been demonstrated. Downstream changes in cellular traits were assessed using MTT, flow cytometry, metastasis and apoptosis assays. These assessments confirmed that the biological behaviors of lung cancer cells were influenced after *SOX2-OT* knockdown. In summary, the present study highlights the oncogenic role of *SOX2-OT* through the regulation of miR-122-3p/FOXO1 and miR-194-5p/FOXA1 pathways.

## Introduction

Lung cancer is the second most common cancer cases that lead to the highest rank of mortality among all cancers worldwide^1,2^. Non-Small Cell Lung Cancer (NSCLC) includes about 80–85 percent of lung cancer cases^1^. Despite advanced in diagnosis and treatment, most lung cancer cases have poor prognoses. Therefore, the recognition of biomarkers as new strategies for early detection and treatment of lung cancer seems essential^2–5^. Long noncoding RNAs (lncRNAs) are mRNA-like transcripts longer than 200 nucleotides without protein-coding capability^6^. Recently, the regulatory function of lncRNAs in various biological and pathological processes has been discovered and reported that LncRNAs dysregulation could affect various biological pathways leading to development of cancers^7,8^. The lncRNA *SOX2-OT* is mapped to human the chromosome 3q26.3 (Chr3q26.3) locus^9^. Recently, it has been made clear that some *SOX2-OT* variants play an oncogenic role in cancer and stem cell-related pathways^10^. The oncogenic role of *SOX2-OT* has been reported in various types of cancer, including lung cancer^11^, breast cancer^12^, ovarian cancer^13^, gastric cancer^14^, glioblastoma^15^, colorectal cancer^16^, hepatocellular carcinoma^17^. One of the lncRNA's functions is gene expression regulation through the sponging to miRNAs that decoy miRNAs, leading to the effect of miRNA downstream mRNA targets^18^. Recently, numerous studies have demonstrated the oncogenic roles of *SOX2-OT* in regulating malignancy traits in cancers by sponging miRNAs, including miR-194-5p in gastric cancer^19^, miR-200 family members in pancreatic ductal adenocarcinoma^20^ and miR-146b-5p in nasopharyngeal carcinoma^21^. Su et al. indicated that *SOX2-OT* knockdown inhibits glioblastoma malignancy behaviors by up-regulating miR-194-5p/SOX3 and miR-122/SOX3, affecting the JAK/STAT signaling pathway^15^. Wang et al. demonstrated that FOXO1 protein functions as a tumor suppressor in the suppression of proliferation, metastasis and induction of apoptosis via miR-122-3p/FOXO1 axis in lung cancer^22^. Also, transcription factor FOXA1 has been demonstrated to be associated with poor overall survival in lung cancer via the miR-194-5p/FOXA1 axis^23^. However, the biological function of *SOX2-OT* in NSCLC progression is not completely clear. Here, we knock down *SOX2-OT* expression by using of RNA interference system and then evaluated the expression of *SOX2-OT* and its regulation effects on miR-122-3p/FOXO1 and miR-194-5p/FOXA1 axes in A549 and Calu-3. as the further aim of the current study, downstream changes in NSCLC cellular behaviors (proliferation, metastasis and apoptosis) examined. In all, our investigation about oncogenic role of *SOX2-OT* via down-regulation of miR-122-3p/FOXO1 and miR-194-5p/FOXA1 axes supports the hypothesis that *SOX2-OT* as an oncogene might be a novel potential target for NSCLC early detection and treatment.

## Results

### Significant knockdown of SOX2-OT and its impacts on miR-122-3p/FOXO1 and miR-194-5p/FOXA1 axes after transfection with *SOX2-OT* siRNAs in A549 and Calu-3 cell lines

**Figure 1:**
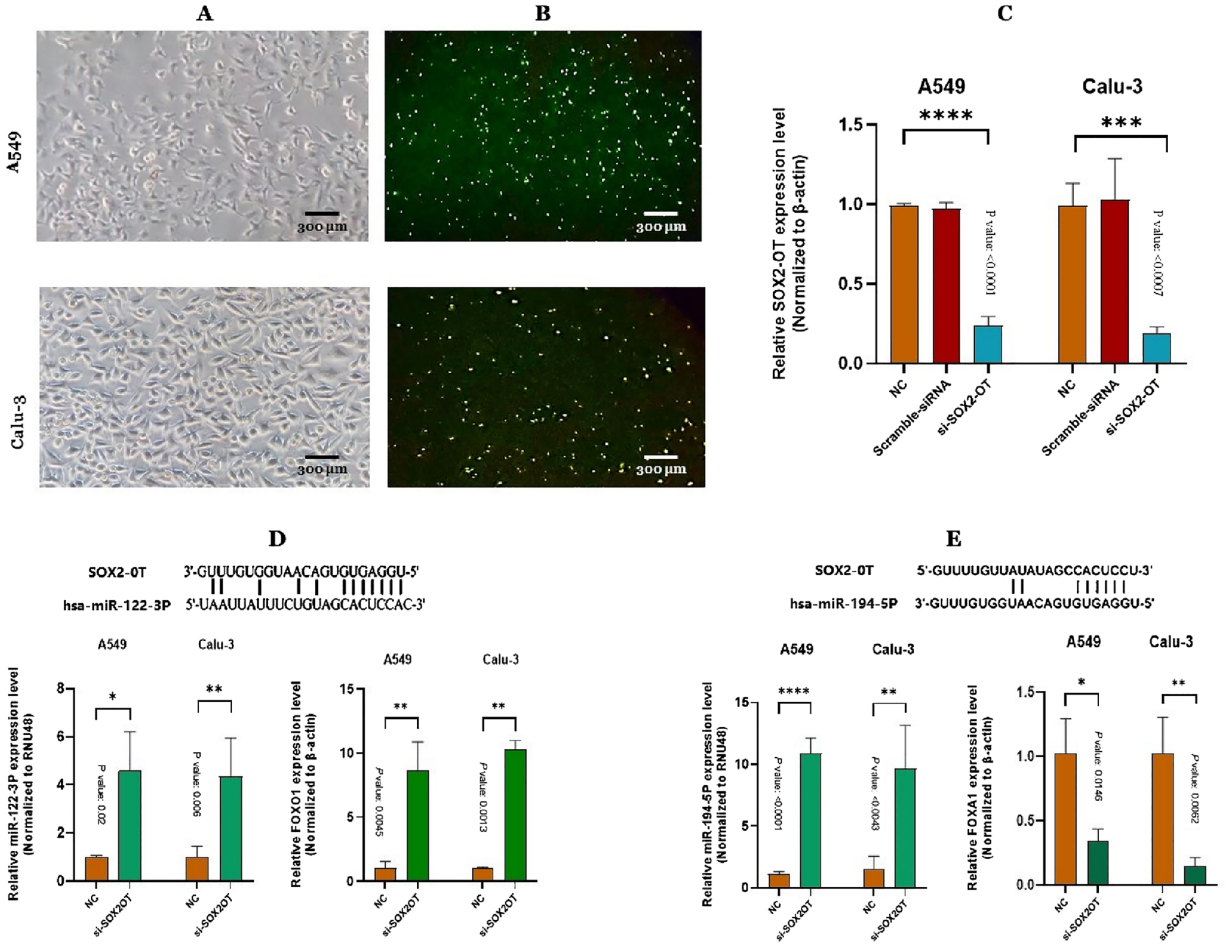
Knockdown of *SOX2-OT* by the siRNAs, down-regulation of miR-122-3p, miR-194-5p and downstream mRNAs (FoxO1 and FoxA1 respectively) relative expression in A549 and Calu-3 cells. (**A**) The light microscopy photographs of siRNA transfected A549 and Calu-3 cells. (**B**) The fluorescent microscope images of transfected cells by dye-labeled siRNA which indicated prospering delivery of siRNA into the cells via Green fluorescent signals. (**C**) The graphs of expression change of *SOX2-OT* after transfection compared with control groups. The control group consist of untreated cells and transfected cells by scrambled siRNA. (**D**) The predicted miR-122-3p binding site in the *SOX2-OT* and graphs of expression change of miR-122-3p and *FOXO1*, its downstream mRNA, after transfection with si-*SOX2-OT* compared with untreated group. (**E**) The predicted miR-194-5p binding site in the *SOX2-OT* and graphs of expression change of miR-194-5p and *FoxA1*, its downstream mRNA, after transfection with si-*SOX2-OT* compared with untreated group. Data are presented as the mean ± SD (*P < 0.05, **P < 0.01, ***P < 0.001, ****P < 0.0001 compared with control groups).

A549 and Calu-3 cell lines were cultured in DMEM medium supplemented with 10% FBS and 1% penicillin-streptomycin, then incubated at 37 °C and 5% CO_2_ in a cell culture incubator. Cell line morphology was observed under light microscopy (Fig. 1A). 48 hours after transfection, relative expression levels of *SOX2-OT*, miR-122-3p, miR-194-5p and miRNAs downstream mRNAs (FOXO1 and FOXA1 respectively) were evaluated by real-time PCR method in cancerous cell lines. Prospering delivery of siRNA into cells was detected via observation of green fluorescent signals after transfection by dye-labeled siRNA in the control group cell lines under the fluorescent inverted microscope (Fig. 1B). Real-time PCR results indicated that expression of *SOX2-OT* in the cells transfected by Si-*SOX2-OT* was reduced significantly compared with untreated cells (****P < 0.0001, ***P < 0.0007 in A549 and Calu-3 cells respectively; Fig. 1C). Whereas, expression of *SOX2-OT* in the control group consisting of untreated and transfected cells with scrambled siRNA were not significantly differed from each other (*P* > 0.05; Fig. 1C). lncRNA *SOX2-OT* binding sites to miR-122-3p and miR-194-5p were taken from starBase v2.0 database (Fig. 1D,E, respectively). Subsequently, relative expression of miR-122-3p/FOXO1 and miR-194-5p/FOXA1 were evaluated in transfected cells by Si-*SOX2-OT* in comparison with untreated cells. Data analysis demonstrated a significant increase of miR-122-3p expression (*P < 0.05, **P < 0.01 in A549 and Calu-3 cells respectively) and a significant increase of FOXO1 expression (**P < 0.01) in transfected cells by Si-*SOX2-OT* compared with untreated cells (Fig. 1D). Meanwhile, miR-194-5p expression was increased (****P < 0.0001, **P < 0.01 in A549 and Calu-3 cells respectively) while FOXA1 expression was decreased significantly (*P < 0.05, **P < 0.01 in A549 and Calu-3 cells respectively) in transfected cells by Si-*SOX2-OT* compared with untreated cells (Fig. 1E). Two-tailed Student's t-test was performed to the evaluation of statistically significant differences between control and treated cells. These results considered that *SOX2-OT* was up-regulated while miR-122-3p/FOXO1 and miR-194-5p/FOXA1 axes were down-regulated in A549 and Calu-3 cells.

### The effects of SOX2-OT knockdown on Cell viability in A549 and Calu-3 cell lines

**Figure 2:**
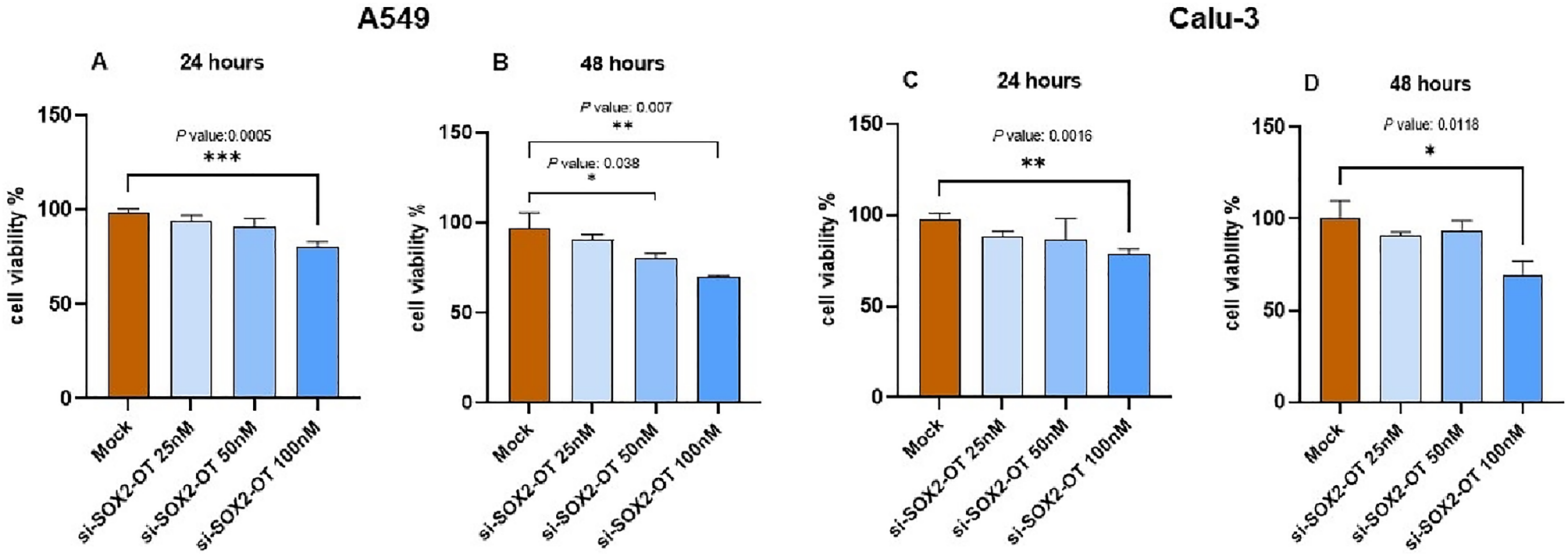
Knockdown effects of *SOX2-OT* on cell viability of A549 and Calu-3 cells. (**A**) The diagrams of MTT assays in A549 cells, 24 h after transfection. (**B**) The diagrams of MTT assays in A549 cells, 48 h after transfection. (**C**) The diagrams of MTT assays in Calu-3 cells, 24 h after transfection. (**D**) The diagrams of MTT assays in Calu-3 cells, 48 h after transfection. Data are presented as the mean ± SD (*P < 0.05, **P < 0.01, ***P < 0.001 compared with untreated cells).

For investigation of *SOX2-OT* knockdown effects on cell viability, the MTT assays were performed 24 and 48 h after transfection with Si-*SOX2-OT* in different concentrations in A549 and Calu-3 cell lines. The analysis of MTT assays results demonstrated that 100nM concentration of Si-*SOX2-OT* in 24 after transfection, 50nM and also 100nM concentration of Si-*SOX2-OT* in 48 h after transfection, significantly affected A549 cell lines compared with untreated cells (***P< 0.001; Fig. 2A and *P< 0.05, **P< 0.01, Fig. 2B, respectively) while, data analysis in Calu-3 cell lines indicated that only 100nM concentration of Si-*SOX2-OT* significantly affected cell viability in 24 and 48 h after transfection compared with untreated cells (**P< 0.01; Fig. 2C and *P< 0.05, Fig. 2D, respectively). Two-tailed Student's t-test was performed to the evaluation of statistically significant differences between untreated and treated cells. These results considered that down-regulation effects of *SOX2-OT* on miRNAs/mRNAs axes could regulate cell viability in lung cancerous A549 and Calu-3 cell lines.

### The effects of SOX2-OT knockdown on metastatic potency in A549 and Calu-3

**Figure 3:**
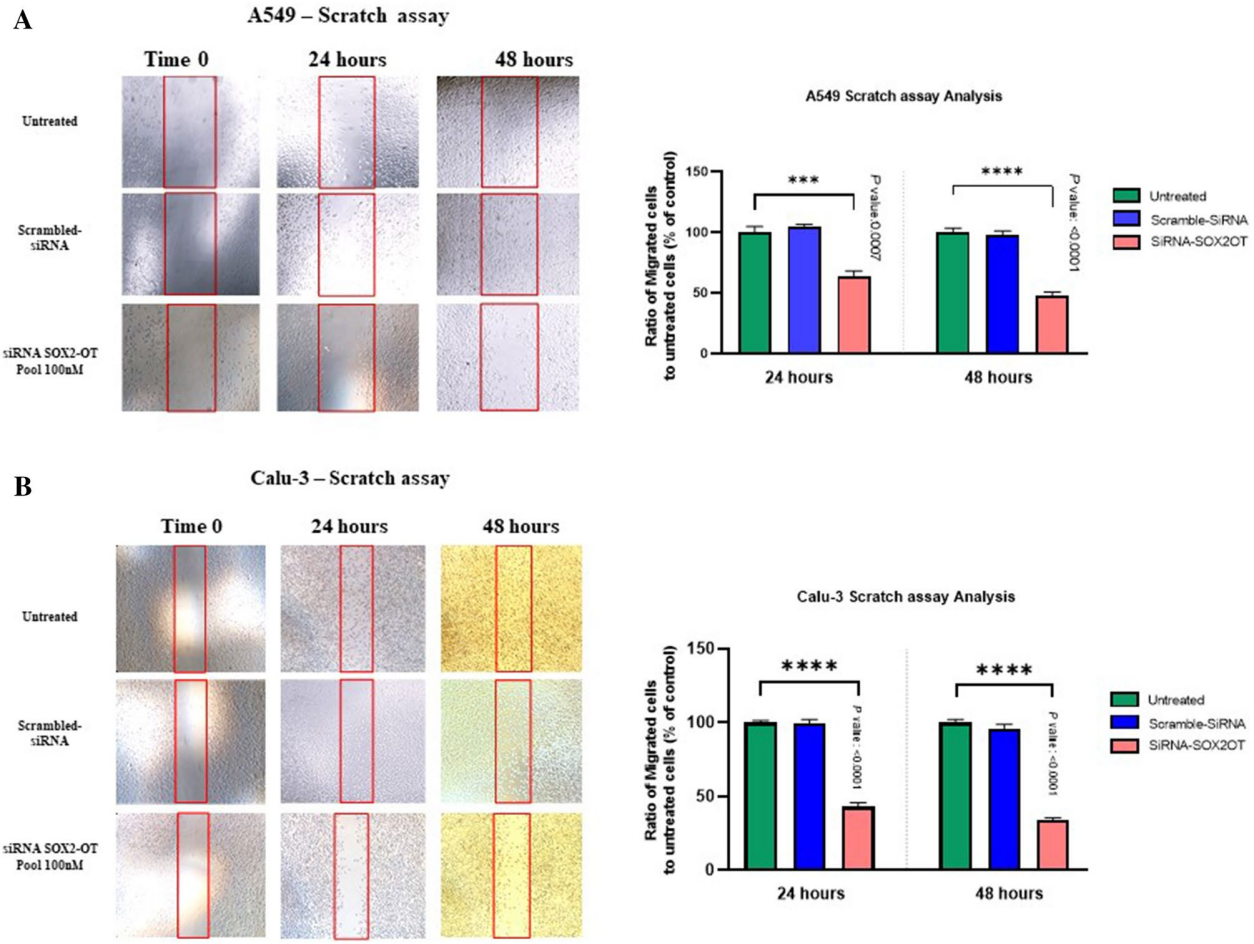
Down-regulation effects of *SOX2-OT* on motility potency of A549 and Calu-3 cell lines. The photographs and diagrams of wound healing assays in scratched control and transfected A549 cells (**A**) and Calu-3 cells (**B**) in time points of 0, 24 and 48 h after scratch with 100 × magnification. The wound edges were demarcated by red lines and the number of migrated cells in the wound sections were counted for each field by ImageJ software. Data are presented as the mean ± SD (***P < 0.001, ****P < 0.0001 compared with control groups).

**Figure 4:**
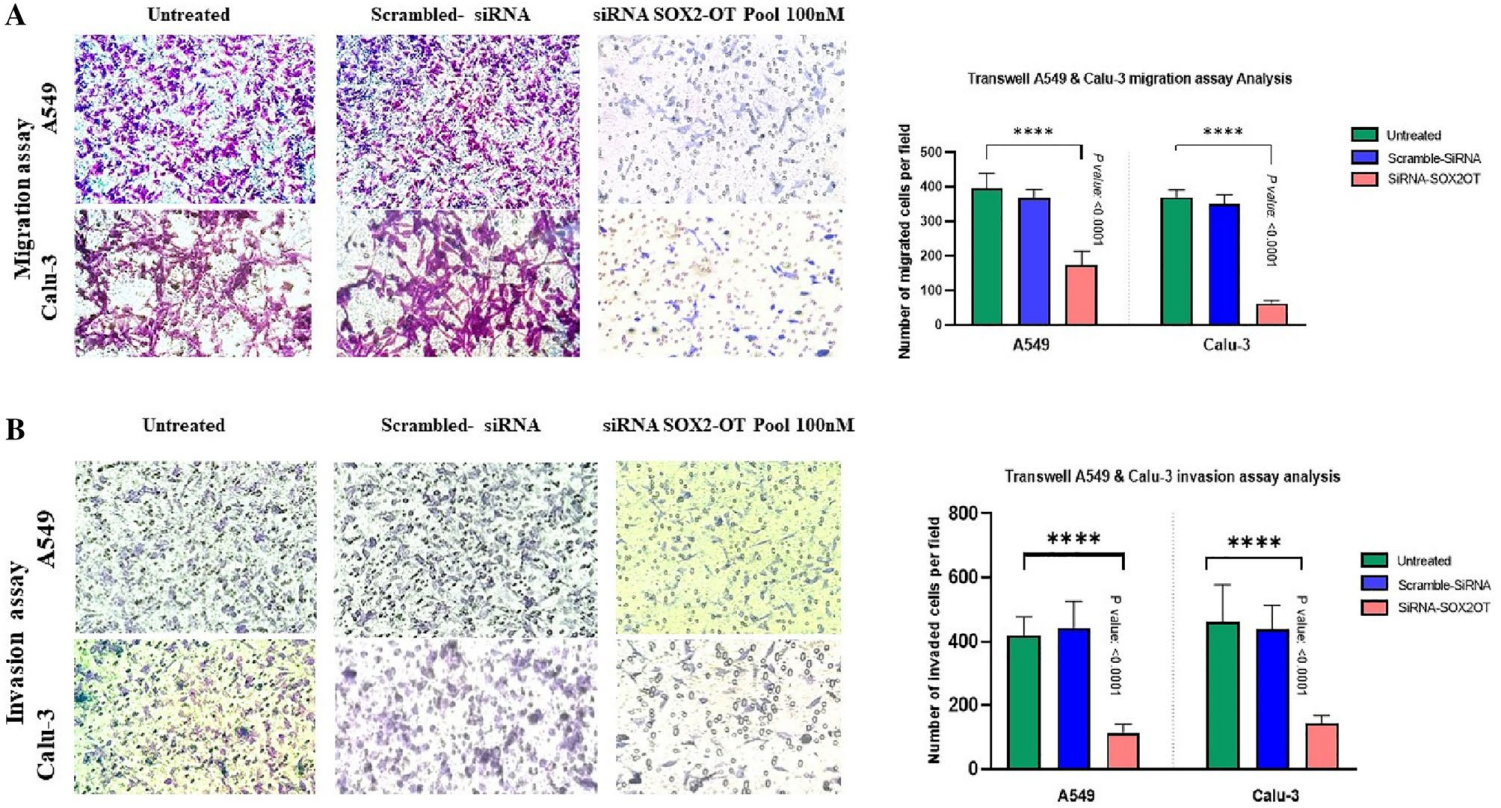
Knockdown effects of *SOX2-OT* on migration and invasion potency of A549 and Calu-3 cell lines. (**A**) The photographs and diagrams of transwell assays for evaluation of *SOX2-OT* Knockdown effects of migration in A549 and Calu-3 cells. (**B**) The photographs and diagrams of transwell assays for evaluation of invasion in A549 and Calu-3 cells. Graphs present with 200 × magnification. Data are presented as the mean ± SD (****P < 0.001 compared with control groups).

For the evaluation of *SOX2-OT* suppression on migration, the wound healing assay was conducted in A549 and Calu-3 cells. The analysis of wound healing assays results demonstrated that 24 and 48 h after transfection with si-*SOX2-OT*, a significance decrease was happened in migrated cell numbers in the wound sections of A549 (***P < 0.001; Fig. 3A and ****P < 0.0001; Fig. 3B) and Calu-3 (****P < 0.0001; Fig. 3B) compared with untreated cells. Subsequently, migration and invasion were evaluated by using transwells and matrigel-coated transwells, respectively. The results indicated a significant decrease in A549 and Calu-3 cell lines (****P < 0.0001; Fig. 4). Two-tailed Student's t-test was performed to evaluate statistically significant differences between untreated and treated cells. These results considered that down-regulation effects of *SOX2-OT* can have oncogenic effects on migration and invasion behaviors in lung cancerous A549 and Calu-3 cell lines.

### The effects of SOX2-OT knockdown on Cell cycle progression in A549 and Calu-3

**Figure 5:**
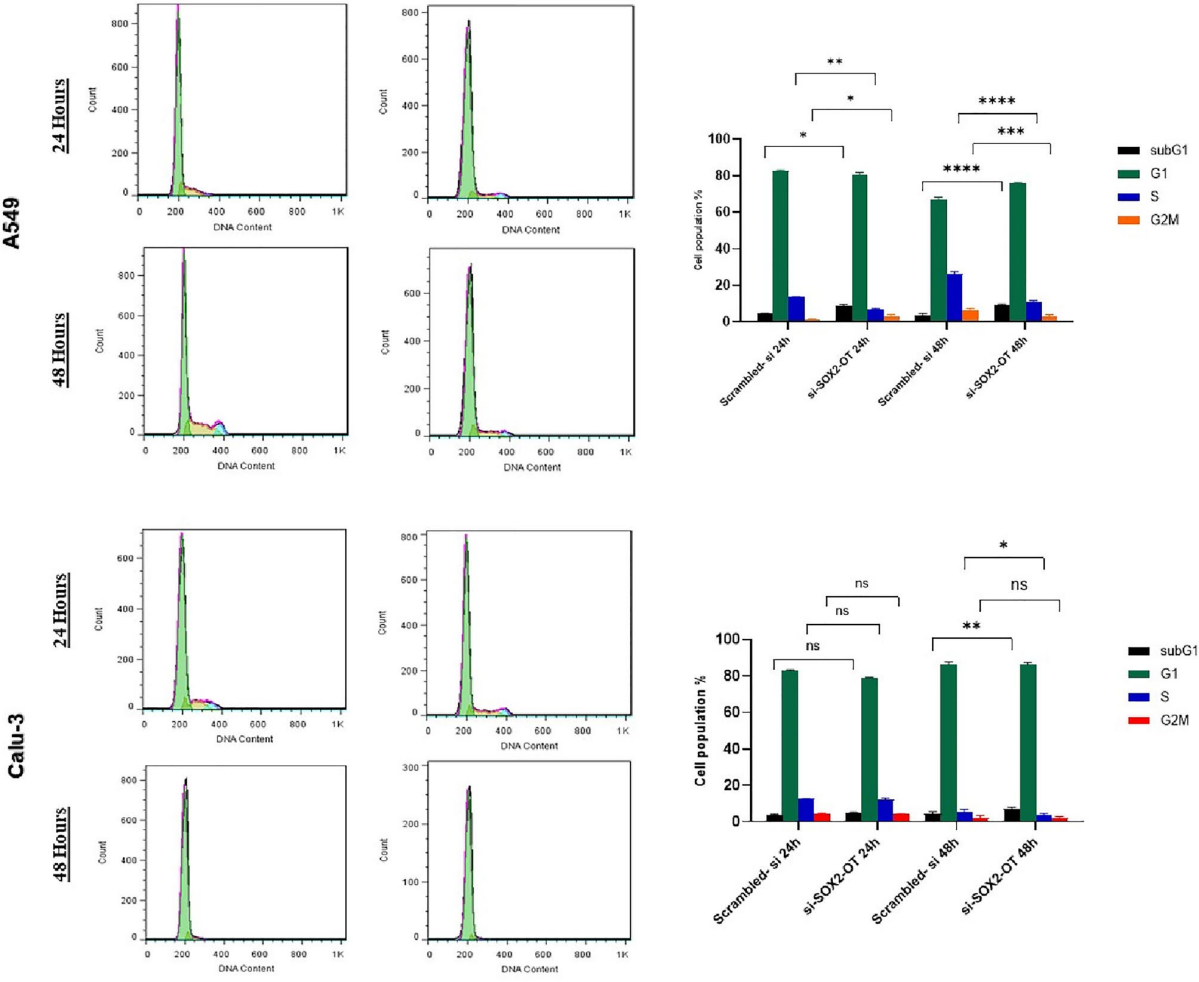
Knockdown effects of *SOX2-OT* on A549 and Calu-3 cells cycle analyzed by flow cytometry. Cell cycle analysis 24 and 48 h after transfection was performed and the histograms and graphs are shown for each cell line separately. cell Population percentages in each phase of cell cycle are presented in graphs. Data are presented as the mean ± SD (*P < 0.05, **P < 0.01, ***P < 0.001, ****P < 0.0001, ns: *P* > 0.05 compared with control groups).

To identify the way of impact of *SOX2-OT* suppression on cell viability via cell cycle arrest or elevation rate of cell death, cell cycle analysis was conducted by flow cytometry 24 and 48 h after transfection with si-*SOX2-OT* and scrambled siRNA in A549 and Calu-3 cells. The data analysis demonstrated a significant elevation of cell population in the sub-G1 phase after *SOX2-OT* knockdown compared with control cells (*P < 0.05, ****P < 0.0001 in A549 cells 24 and 48 h after transfection, respectively). Also, A549 cell populations in the G2/M phase were decreased after *SOX2-OT* suppression compared with control cells (*P < 0.05, ***P < 0.001 in A549 cells 24 and 48 h after transfection, respectively). Although, in the Calu-3 cell line population in sub-G1 and G2/M at the time point of 24 h after transfection, significant variation was not observed (#*P *> 0.05) at time point 48 h after transfection, significant elevation of cell population at sub-G1 phase was observed (**P < 0.01) compared with control cells. Meanwhile, a significant decrease of cell population at the S phase at points 24 and 48 h in A549 cells (**P < 0.01, ****P < 0.0001) and 48 h after transfection in Calu-3 cells (*P < 0.05) were observed compared with control cells. Overall, the results investigated that *SOX2-OT* knockdown could affect cell viability by cell cycle arrest and cell death rate (Fig. 5).

### The effects of SOX2-OT knockdown on cell apoptosis

**Figure 6:**
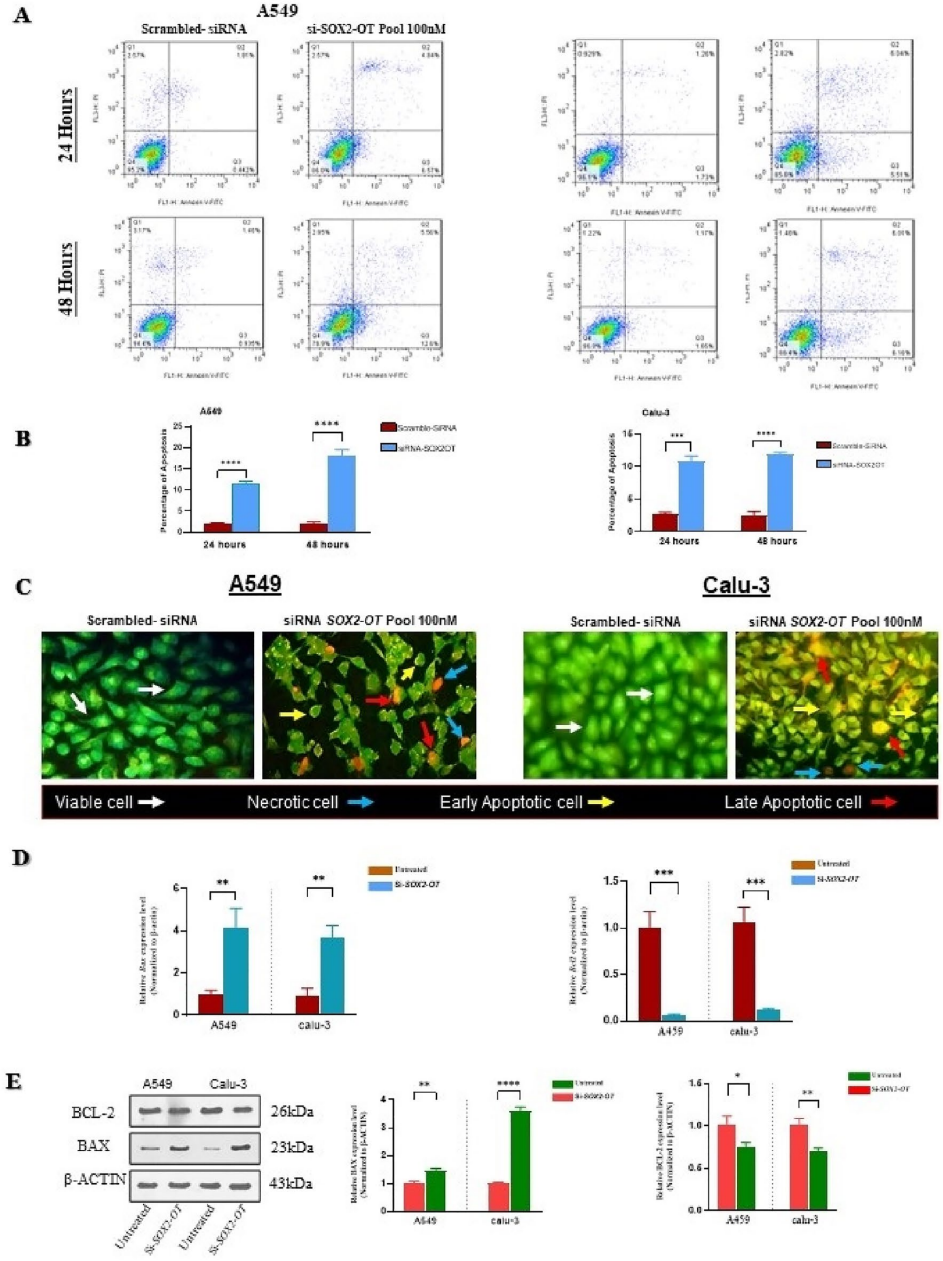
Knockdown of *SOX2-OT* effects on impression of apoptosis in A549 and Calu-3 cells. (**A**) The flow cytometry analysis after Annexin V/FITC/PI staining in A549 and Calu-3 cells, 24 and 48 h after transfection. In the diagram, quarters of Q1, Q2, Q3, Q4 represent dead/necrotic cells, late apoptosis, early apoptosis and live cells, respectively. (**B**) The diagrams apoptosis rate in A549 and Calu-3 cells, 24 and 48 h after transfection. (**C**) Acridine orange and ethidium bromide staining in A549 and Calu-3 cells, 48 h after transfection. (**D**) The graphs of Relative expression of *Bax* and *Bcl2* genes after transfection with si-*SOX2-OT* compared with untreated group. (**E**) Representative western blot and the graphs of quantification of expression of BAX and BCL-2 after transfection with si-*SOX2-OT* compared with untreated group. The original image can be obtained in Supplementary Fig. S1 Data are presented as the mean ± SD (*P < 0.05, **P < 0.01, ***P < 0.001, ****P < 0.0001 compared with control groups).

Cell cycle analysis indicated that cell viability, proliferation and induced cell death were impressed due to *SOX2-OT* suppression. Therefore, flow cytometric analysis after Annexin V/FITC/PI staining was performed to evaluate apoptosis and necrosis rate in A549 and Calu-3 cells, 24 and 48 h after transfection. Results of flow cytometry indicated that the apoptosis rate in transfected cells was increased. So that in A549 cells, late apoptosis rates in control and *SOX2-OT* suppressed cells were 1.81% and 4,84%, 24 h after transfection, respectively. While a late apoptosis rate of 1.34% in control cells was increased to 5.56% in *SOX2-OT*-suppressed cells 48 h after transfection. Data analysis in Calu-3 cells indicated that late apoptosis rates in control and *SOX2-OT*-suppressed cells were 1.37% and 6.04%, 24 h after transfection, respectively. While a late apoptosis rate of 1.25% in control cells was increased to 6.01% in *SOX2-OT* suppressed cells 48 h after transfection (Fig. 6A). Overall, flow cytometry analysis data demonstrated that a significant increase in apoptosis rate was happened in the cells with *SOX2-OT* suppression (***P < 0.001, ****P < 0.0001; Fig. 6B) compared with control groups.

In the next step, for visualization of apoptosis and after transfection of the cell lines, AO/EB staining was performed. AO/EB staining indicated an event of apoptosis in the cells that were transfected by si-*SOX2-OT* as a result of apoptosis inspiration. Cellular morphology differed in cells with *SOX2-OT* suppression compared with control cells. The normal cells stained only with AO and thus took green color. The normal cells had normal nucleus morphology and integral cell membrane. In early apoptotic cells, chromatin condensed and the cell membrane started to blebbing. The early apoptotic cells had a granular crescent nucleus. In the late apoptotic cells, due to the formation of multiple irregular dense spots, apoptotic bodies, nucleus stain with EB and cells are seen orange (Fig. 6C).

Subsequently, for evaluation of induction of apoptosis in the cells after *SOX2-OT* suppression, relative expression levels of Bax and Bcl2 genes, that are involved in cell death, were investigated. Data analysis of Bax and Bcl2 genes expression after transfection with si-*SOX2-OT* investigated significant up-regulation of Bax expression and significant down-regulation of Bcl2 expression in A549 and Calu-3 cells (**P < 0.01, ***P < 0.001; Fig. 6D) compared with control groups.

Eventually, for evaluation of induction of apoptosis in the cells after *SOX2-OT* suppression, relative expression levels of Bax and Bcl2 proteins were investigated with western blot assay. The Western blot assay results corroborated the qRT-PCR findings for BAX and BCL2 expression levels after si-*SOX2-OT* knockdown, indicating apoptosis occurrence (Supplementary Fig. S1). Data analysis of Bax and Bcl2 proteins expression level after transfection with si-*SOX2-OT* supported significant up-regulation of Bax expression and significant down-regulation of Bcl2 expression in A549 and Calu-3 cells ((*P < 0.05, **P < 0.01, ***P < 0.001, ****P < 0.0001; Fig. 6E) compared with control groups.

## Discussion

Recently, studies have demonstrated that dysregulation of lncRNAs plays critical roles in the development of most cancers, including NSCLC^24,25^. The oncogenic role of *SOX2-OT* has been established through numerous studies in cancer research. High expression of lncRNA *SOX2-OT* is associated with poor survival rates in lung cancer patients^26^. Similarly, evidence shows that *SOX2-OT* expression is significantly higher in NSCLC tissues and serum samples compared to normal controls^27,28^. Marie et al. reported that the expression of *SOX2-OT* variants 4 and 7 was higher in NSCLC tumors, particularly in lung squamous cell carcinoma^29^. Teng et al. discovered that *SOX2-OT* expression is linked to specific clinical pathological parameters, including tumor size and lymph node metastasis^30^. Also, Li et al. (2018) examined the expression levels of *SOX2-OT* NSCLC tissues and their correlation with clinical features and prognosis. The study revealed that high expression levels of *SOX2-OT* were significantly associated with poor overall survival and disease-free survival in NSCLC patients^31,32^. Therefore, in the present study, we knock down *SOX2-OT* by using of RNA interference system and investigated that *SOX2-OT* suppression not only could inhibit malignancy traits including proliferation, migration and invasion but also could induce apoptosis in the A549 and Calu-3 cell lines. The results of the present study demonstrated that lncRNA *SOX2-OT* as an oncogene enhances NSCLC biological behaviors.

Recently, studies found that lncRNAs could act as molecular sponges, decoy miRNAs and regulate cellular pathways with the effect of miRNA downstream mRNA targets^18,33^. *SOX2-OT* has been recognized as an oncogene in various cancer by sponging miRNAs, including, miR-194-5p^19^, miR-200 family members^20^ and miR-146b-5p^21^, miR146b-5p^25^, miR-194-5p and miR-122^15^. Su et al. found that *SOX2-OT* knockdown inhibited glioblastoma stem cell behaviors including proliferation, migration and invasion and induced apoptosis via the miR-194-5p/miR-122-SOX3-TDGF-1 pathway^15^. Therefore, as the aim of present study after *SOX2-OT* knockdown, we evaluated the expression of miR-194-5p and miR-122-3p in A549 and Calu-3 cell lines. We found that after *SOX2-OT* suppression, expression levels of miR-194-5p and miR-122-3p elevated significantly compared with untreated cell lines. As shown in previous studies, we found miR-194-5p and miR-122-3p acted as tumors suppressor miRNAs in NSCLC so that, the A549 and Calu-3 cell lines with increased expression levels of miR-194-5p and miR-122-3p had significantly low levels of metastasis, proliferation and increased apoptosis rate compared with untreated A549 and Calu-3 cell lines. Furthermore, previous studies have indicated that FOXO proteins, as tumor suppressors regulate cellular functions, including cell cycle progression, apoptosis, DNA damage repair and energy metabolism and have been reported that FOXO overexpression is associated with the reduction of tumor growth, control of cellular homeostasis and stability^34,35^. Wang et al. found that FOXO1 protein function as a tumor suppressor for inhibition of proliferation, metastasis and induction of apoptosis via miR-122-3p/FOXO1 axis in lung cancer^22^. Here, we knock down *SOX2-OT* expression by using of RNA interference system and then the expression of *SOX2-OT*, miR-122-3p and FOXO1 were evaluated. Interestingly, we found significantly increased expression levels of miR-122-3p and FOXO1 after *SOX2-OT* knockdown conversely with decreased *SOX2-OT* expression. As shown in previous studies, dysregulation of Bax and Bcl-2 expression levels were indicated in various cancers, so that Bcl-2 as a stimulator cell cycle progression, had high-level expression^36–39^. In comparison, Bax as a pro-apoptotic molecule had a low-level expression ratio in various cancers^40,41^. Therefore, after investigation of the significantly decreased expression level of FOXO1 after *SOX2-OT* knockdown, the downstream effect on Bax and Bcl-2 expression levels was evaluated. followed by results based on AO/EB staining assay and a significantly increased population of sub-G1 phase in results of cell cycle analysis assay that indicated induced apoptosis, Bcl-2 expression levels were increased but Bax expression levels decreased significantly after *SOX2-OT* knockdown in A549 and Calu-3 cell lines. Our findings confirmed that lncRNA *SOX2-OT* as an oncogene enhances NSCLC cells survival via inhibition of the miR-122-3p/FOXO1 axis. Deutsch et al. demonstrated that transcription factor FOXA1 as an oncogene has been associated with metastasis and poor overall survival in lung cancer^42^. Another study explained that FOXA1 had an association with the propensity to metastasis in NSCLC cells with the promotion of epithelial to mesenchymal transition (EMT) in NSCLC^43^. Zho et al. indicated that transcription factor FOXA1 as an oncogene has been associated with metastasis and poor overall survival in lung cancer and miR-194-5p inhibited proliferation, invasion and migration, and enhances the chemosensitivity in NSCLC by targeting FOXA1^21^. In this study, after *SOX2-OT* knockdown, evaluated miR-194-5p and FOXA1 expression levels. Interestingly, we found that increased expression of miR-194-5p and decreased FOXA1 expression after *SOX2-OT* knockdown, approved the oncogenic role of FOXA1. Our study investigated that FOXA1 was under the influence regulatory role of miR-194-5p and after *SOX2-OT* knockdown, as a result of suppression FOXA1, a significant decrease in migration and invasion rate of A549 and Calu-3 cell lines occurred in the transwell assay. on the other hand, a significant decrease in the population of S phase in the results of cell cycle analysis assay was observed after *SOX2-OT* knockdown in our study. In accordance with previous studies^15,21^, our funding conformed to enhancing role of FOXA1 in proliferation of NSCLC cell lines. Overall, we found that *SOX2-OT* knockdown inhibited NSCLC behaviors including proliferation, migration and invasion, while inducing apoptosis through the regulation of miR-122-3p/FOXO1 and miR-194-5p/FOXA1 axes.

In conclusion, our study indicated that lncRNA *SOX2-OT* enhances malignancy behaviors of NSCLC cell lines as an oncogene via sponging to tumor suppressor miR-122-3p and miR-194-5p resulting in dysregulation downstream pathways. Therefore, these findings suggested that lncRNA *SOX2-OT* could consider a novel new biomarker for early detection and efficient treatment of NSCLC.

## Materials and methods

### Cell culture and siRNA transfection

Two human lung adenocarcinoma cell lines (A549 and Calu-3) were purchased from Pasture Institute (Tehran, Iran). The cell lines were cultured in Dulbecco’s Modified Eagle’s Medium (DMEM, Gibco) supplemented with 10% fetal bovine serum (FBS, Gibco) and 1% penicillin–streptomycin at 37 °C in a 98% humidified atmosphere with 5% CO_2_ cell culture incubator (binder). The cell lines were transfected with siRNA against *SOX2-OT* (si- *SOX2-OT*) with 19 nucleotide length and Scrambled siRNA as a negative control (purchased from Qiagen Company). siRNA targeting *SOX2-OT* did not have modification as fluorescein dye, therefore a 5ʹ-Fluorescein (6 FAM) dye-labeled siRNA to be used for detection and visible delivery of siRNA in transfection of cell lines in the control groups under the fluorescent inverted microscope. For siRNA transfection, 3 × 105 cells were seeded in each well of 6-well tissue culture plates for an additional 1 day prior to siRNA transfection. 2 hours before transfection media was replaced with reduced FBS (5%) and free antibiotics media. Then transfection was performed at 70% confluency of cell lines by Lipofectamine 2000 (Invitrogen; Thermo Fischer Scientific. Inc., Waltham, MA, USA) according to the manufacturer’s instructions. All experiments were performed in the thrice independent test.

### RNA extraction, quality control and cDNA synthesis

48 hours after transfection, the total RNA from untreated and transfected cell lines was extracted by RNX-Plus solution (Sinaclon, Iran) according to the manufacturer’s procedure manual. The quantity and quality of the extracted RNA were controlled by NanoDrop microspectrophotometer at 260 nm (Thermo Fisher Scientific, US) and electrophoresis on agarose gel respectively. Following this, 2 µg of total RNA after treatment by DNaseΙ (Thermo Fisher Scientific, Inc) was reverse transcribed into cDNA by using an AddScript cDNA Synthesis kit (AddBio, Korea). For measuring of *SOX2-OT* and other mRNA expression levels, oligo dT and random hexamer primers were utilized for the synthesis of the first strand of cDNA. For analysis of miR-122-3P and miR-194-5p expression levels, exclusive cDNA for each miRNA was synthesized from the specific Stem-loop RT primer. Stem-loop RT primers were designed according to Chen et al.^44^.

### Quantitative real-time PCR (qRT-PCR)

The qRT-PCR was performed by BioFACTTM 2X Real-Time PCR Master Mix (BIOFACT, South Korea) in the SYBR Green chemistry method. The qRT-PCR programs were performed in the Mic qPCR Cycler system with specific programs for each primer pair. Specific primers listed in Table 1 were applied in qRT-PCR. *SOX2-OT* and mRNA expression levels were normalized by β-actin as an endogenous control. In addition, SNORD U48 was utilized for miRNA expression level normalization. The relative studied genes expression level was measured by using the 2^−ΔΔCt^ method.

**Table 1:** Real-time PCR Oligonucleotide primers used for relative expression analysis.

Target gene	Primer (5ʹ to 3ʹ)
SOX2-OT	F: GCTCGTGGCTTAGGAGATTG R: CTGGCAAAGCATGAGGAACT
FOXA1	F: GCAATACTCGCCTTACGG R: TACACACCTTGGTAGTACG
FOXO1	F: CGCAGATTTACGAGTGGATGG R: CACTCTTGCCTCCCTCTGG
β-actin	F: AGACGCAGGATGGCATGGG R: GAGACCTTCAACACCCCAGCC
122-3p	F: TTCACGCAACGCCATTATCAC
194-5p	F: AACGCAGTGTAACAGCAACTC

### MTT assay for cell viability

Evaluation of cell metabolic activity for indication of cell viability was performed by 3-(4, 5-dimethylthiazol-2-yl)-2 5-diphenyl-tetrazolium bromide (MTT) colorimetric assay (Sigma-Aldrich, St. Louis, Mo, USA). Briefly, 1×104 cells were seeded per well of 96-well plates in triplicate repeat and transfected by varied concentrations of *SOX2-OT*-siRNAs. After 24 and 48 h, 15 μl MTT solution (5 mg/ml) was added to each well and incubated for 4 h in the incubator. Then, after replacement of media and aimed at dissolve of formazan crystals, 150 μl dimethyl sulfoxide (DMSO, Sigma-Aldrich) was added. Subsequently, the absorbance value of each well was measured at 570 nm by using a microplate photometer (LabSystem Multiscan). The experiment was performed in the thrice independent test.

### Wound healing assay

The two-dimensional cell migration was evaluated with the wound healing assay. 24 hours after transfection, the media was changed with 0.5% FBS overnight to minimize cell proliferation. When the confluency reached 80–90%, cell monolayers were wounded with a yellow tip. After washing cells twice with sterile phosphate-buffered saline (PBS) and adding fresh media, the plate was incubated in the incubator. The wounded region was imaged with 40× magnification by an inverted microscope immediately, 24 and 48 h later. The migrated cells in the scratch regions were counted using ImageJ software, version 1.4.1, for identical fields of images.

### Transwell assay

The cell migration and invasion ability were evaluated by using of transwell chambers with 8.0 μm pore size. Briefly, 24 h after transfection, 5 × 104 cells were suspended in 250 μl serum-free media and seeded into the upper chamber of the inserts in the transwell plates (SPL). Then, 750 μl media with 10% FBS was added into the lower chamber as a chemoattractant factor. Plates were incubated for 48 h in a cell culture incubator. Then, the remainder cells on the upper surface of the transwells were scraped off using a cotton swab, whereas the migrated cells to the bottom side were fixed with 3.7% paraformaldehyde for 20 min and stained with Giemsa solution for 15 min. For invasion assay, the upper 8 μm chambers were coated with Matrigel Matrix (Life Sciences, USA) and dried for 4 h in the incubator. The next day, 5 × 104 cells were seeded on the Matrigel-coated chambers and the procedure was done as mentioned above method for the migration assay. The number of penetrated cells was counted in six random selected fields in the images with 200× magnification imaged by an inverted microscope.

### Cell cycle analysis

Cells in different phases of the cell cycle were investigated by flow cytometer. 24 and 48 h after transfection the treated cells with siRNAs (100 nM) were washed with PBS, harvested and then fixed with cold ethanol (70%). Then the cells were washed twice with PBS, resuspended in the PI/Triton X-100 staining solution (Triton X-100 (0.1% [v/v]), 10 μg/ml propidium iodide and 100 μg/ml DNase-free RNase A in PBS), and incubated for half an hour in the dark condition at room temperature. Eventually, progression in different phases of the cell cycle was evaluated by BD FACSCalibur flow cytometer (BD Biosciences, USA) and analyzed by using the FlowJo software (Tree Star, Inc).

### Apoptosis assay by flow cytometry

The cellular apoptosis was evaluated by Annexin V/FITC/PI staining in flow cytometry. 24 and 48 hours after transfection the treated cells with siRNAs (100 nM) were washed with PBS and harvested. Then, the suspended cells in PBS were stained with Annexin V/FITC/PI apoptosis detection kit (BD Biosciences, USA) for 15 min in the dark condition at room temperature according to the manufacturer’s instruction. By dual staining, detection of early-stage apoptosis (Annexin-positive cells) and late-stage apoptosis/necrosis (PI-positive cells) became possible. Subsequently, the stained cells were immediately evaluated by a BD FACSCalibur flow cytometer (BD Biosciences, USA) and the acquired data were analyzed using the FlowJo software (tree stat, INC).

### Acridine orang/ethidium bromide (AO/EB) fluorescent staining assay

Dual fluorescent staining AO/ET (Sigma-Aldrich) was performed for visualizing apoptosis in the treated cells with reduced expression of *SOX2-OT* compared to the control groups. After washing the cells with PBS, they were stained with 5 μg/ml AO and 5 μg/ml EB in 1X PBS. Then, the stained cells were washed with PBS, observed and imaged under a fluorescence microscope. For the distinction between viable, necrotic and apoptotic cells, penetration of different dyes into intact cell membranes was considered.

### Western blot assay

Cells were lysed applying RIPA buffer (Beyotime) containing protease inhibitors (Roche) and the proteins were extracted from the cells and separated by size using SDS-PAGE (sodium dodecyl sulfate-polyacrylamide gel electrophoresis). The separated proteins were then transferred from the gels onto PVDF membranes. The membranes were blocked with a 3% bovine serum albumin solution at room temperature for 1 h. Next, membranes were incubated overnight in the primary antibodies’ solution (anti-BAX; Santa Cruz Biotechnology, INC, sc-7480 and BCL-2; Santa Cruz Biotechnology, INC, sc-492) with 1:1000 dilution at 4 °C. Afterward, the membranes were rinsed 3 times for 5 min with TBST and incubated in the HRP-conjugated secondary antibody solution (Santa Cruz Biotechnology, INC, sc-2357) for 1 h at room temperature and rinsed the blots 3 times for 5 min with TBST. Finally, the chemiluminescent substrate was applied to the blot following to the manufacturer’s recommendation, and the chemiluminescent signals were captured using a CCD camera-based imager (Thermo Fisher Scientific, US) and quantified using ImageJ software, version 1.4.1 and were normalized to the β-actin bands as control.

### Statistical analysis

GraphPad Prism 9.4.0 (GraphPad Software Inc, San Diego, CA, USA) was applied for data analysis. The data are shown as mean ± standard deviations (S.D) to be obtained from the thrice independent test. Two-tailed Student's t-test and two-way analysis of variance (ANOVA) were performed for Analyze and comparison between two groups and multiple groups, respectively. The *P*-value of < 0.05 indicated a statistically significant difference in the results of the Analysis.

## Supplementary Information

Supplementary Figure S1.

## Data Availability

The datasets used and analyzed during the current study are available from the corresponding author on reasonable request.
