# Supplementary material for: Long non-coding RNA SOX2-OT enhances cancer biological traits via sponging to tumor suppressor miR-122-3p and miR-194-5p in non-small cell lung carcinoma
Source: Sci Rep. 2023 Jul 31;13:12371. doi: 10.1038/s41598-023-39000-0 (PMC10390639; doi:10.1038/s41598-023-39000-0)
Supplement: Supplementary file 1 — Supplementary Figure S1. [file 41598_2023_39000_MOESM1_ESM.pdf]

Long non-coding RNA *SOX2OT* enhances cancer biological traits via sponging to tumor suppressor miR-122-3p and miR-194-5p in non-small cell lung carcinoma

Fatemeh Dodangeh[1], **Zahra Sadeghi**[1], **Parichehr Maleki**[1], **and Jamshid Raheb**[1,*]

[1]Department of Molecular Medicine, National Institute of Genetic Engineering and Biotechnology, Tehran, Iran.
[*]Jam@nigeb.ac.ir

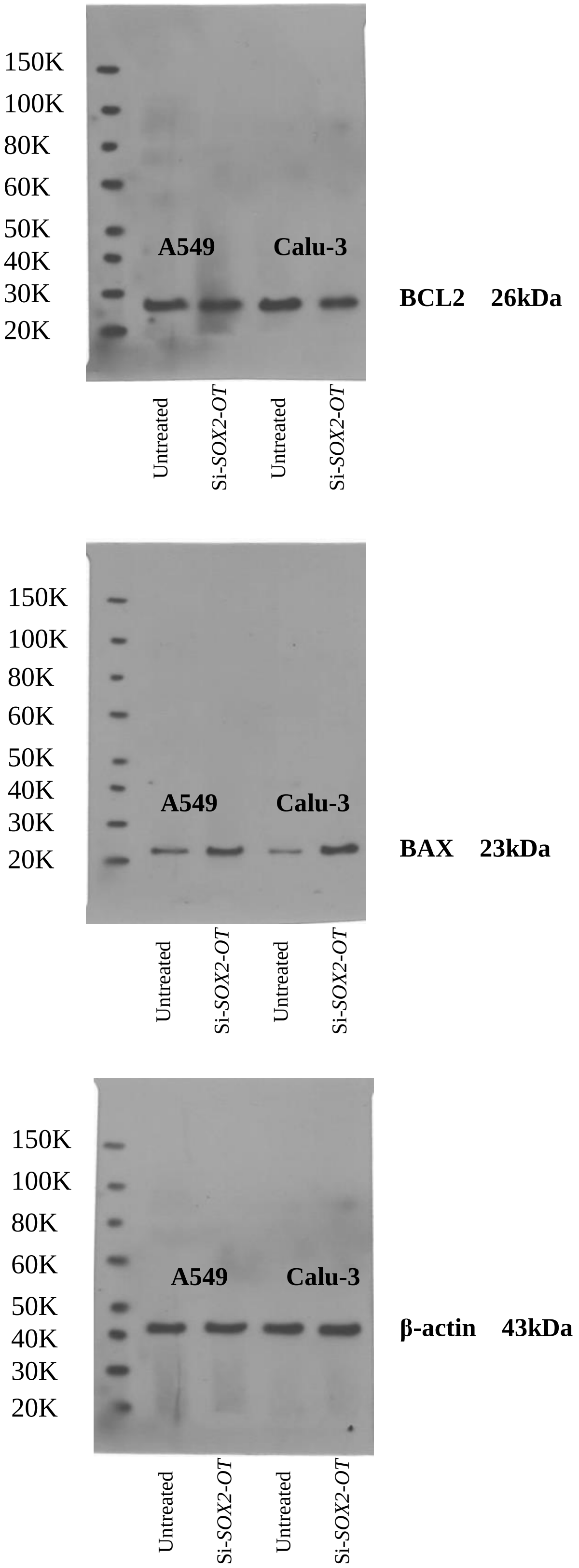

Supplementary Fig. S1. The uncropped original data of Fig. 6E. western blot of expression of BAX and BCL-2 after transfection with si-*SOX2-OT* compared with untreated group.
